# QSAR analysis of VEGFR-2 inhibitors based on machine learning, Topomer CoMFA and molecule docking

**DOI:** 10.1186/s13065-024-01165-8

**Published:** 2024-03-30

**Authors:** Hao Ding, Fei Xing, Lin Zou, Liang Zhao

**Affiliations:** 1 Department of Ultrasound, Shengjing Hospital of China Medical University, Shenyang, 110004 Liaoning China; 2 Hepatobiliary and Splenic Surgery Ward, Department of General Surgery, Shengjing Hospital of China Medical University, Shenyang, 110004 Liaoning China; 3 Department of Oncology, Shengjing Hospital of China Medical University, Shenyang, 110004 Liaoning China; 4 Medical College of Guangxi University, Nanning, 530004 Guangxi China

**Keywords:** VEGFR-2, Structure–activity relationship model, Machine learning, Molecular docking

## Abstract

VEGFR-2 kinase inhibitors are clinically approved drugs that can effectively target cancer angiogenesis. However, such inhibitors have adverse effects such as skin toxicity, gastrointestinal reactions and hepatic impairment. In this study, machine learning and Topomer CoMFA, which is an alignment-dependent, descriptor-based method, were employed to build structural activity relationship models of potentially new VEGFR-2 inhibitors. The prediction ac-curacy of the training and test sets of the 2D-SAR model were 82.4 and 80.1%, respectively, with KNN. Topomer CoMFA approach was then used for 3D-QSAR modeling of VEGFR-2 inhibitors. The coefficient of q2 for cross-validation of the model 1 was greater than 0.5, suggesting that a stable drug activity-prediction model was obtained. Molecular docking was further performed to simulate the interactions between the five most promising compounds and VEGFR-2 target protein and the Total Scores were all greater than 6, indicating that they had a strong hydrogen bond interactions were present. This study successfully used machine learning to obtain five potentially novel VEGFR-2 inhibitors to increase our arsenal of drugs to combat cancer.

**Supplementary Information:**

The online version contains supplementary material available at 10.1186/s13065-024-01165-8.

## Introduction

Hepatocellular carcinoma (HCC) originates from hepatocytes and is a malignant tumor with the pathological characteristics of cancer cells arranged in substantial masses [1]. The number of deaths from liver cancer increased significantly every year, and the 5-year survival rate ranges from 12 to 35% depending on how early it is detected [2, 3]. In China, about 383,000 people die from liver cancer every year, and this accounts for about 51% of the world’s total [4]. The fatality rate also ranks second in the cause of death from malignant tumors in China, and the recurrence rate is high with poor prognosis [4, 5].

Vascular endothelial growth factor receptors (VEGFR) including VEGFR-1, VEGFR-2 andVEGFR-3 are members of the tyrosine kinase receptor superfamily. VEGFR-2 is widely expressed in epithelial cells, smooth muscle tissue, electrically excited cells and some tumor cells [6]. It is also highly expressed in cancer cells and is mainly involved in tumor growth and proliferation [6]. The malignant proliferation of hepatoma cells not only depends on their own rapid growth characteristics, but is also related to the local microenvironment and angiogenesis [7, 8]. Studies also show that hepatoma cells pro-mote angiogenesis by secreting VEGF-A and express VEGFR. In addition, they activate intracellular VEGFR-2 on tumor cell membranes in order to promote growth [9, 10]. Hence, VEGFR-2 could be considered as a key drug target for the treatment of liver cancer.

VEGFR-2 inhibitors are generally classified into the following categories according to their binding mode: (a) Type I kinase inhibitors, such as sunitinib and brivanib alaninate, whose heterocycles competitively occupy the hydrophobic cavity with hydrophobic forces, instead of ATP [11, 12]. (b) Novel type I kinase inhibitors, including lenvatinib, fruquintinib and axitinib, each of which has an additional chemical fragment based on the structure of type I kinase inhibitor. These can interact with narrow cavity of the pocket at Asp1046 and Glu885 through hydrogen bonds [13–15]. (c) In addition to forming hydrogen bonds with the amino group, type II kinase inhibitors including, sorafenib, tivozanib and cabozantinib, which can occupy aromatic rings of the two hydrophobic cavities [16–18]. (d) Novel type II kinase inhibitors, including ponatinib, which can introduce a structural fragment to the aromatic ring of the type II kinase inhibitor to occupy the outer portion of hydrophilic cavity II [19]. However, the weak selectivity against VEGFR-2 kinase inhibitors may also lead to adverse effects such as skin toxicity, gastro-intestinal reactions and hepatic impairment. Hence, there is a need to find novel and effective VEGFR-2 inhibitors for use in cancer treatment. Due to the difficulties in drug development, molecular docking, structure–activity relationships/quantitative-structure–activity relationships and other computer-aided drug research are gradually playing an important role in the field of drug design [20–23].

In this study, machine learning, Topmer CoMFA and molecule docking approaches were used to build two-dimensional/structure–activity relationships (2D-SAR), three-dimensional quantitative-structure–activity relationships (3D-QSAR) and VEGFR-2 inhibitors-receptor interaction models were used to find potentially new VEGFR-2 inhibitors.

## Materials and methods

### Data preparation

The process of selecting training and validation sets in a study involves choosing subsets of data from the overall dataset to build and assess a predictive model. The selection is typically guided by principles that ensure the model’s generalizability and effectiveness. Random sampling is employed to ensure that both the training and validation sets are representative of the overall dataset. Here, for the 2D-SAR investigation, a training set consisting of 243 inhibitors and 275 non-inhibitors was randomly chosen (refer to Additional file 1:Training set for 2D-SAR). Similarly, a test set was established, comprising 72 inhibitors and 71 non-inhibitors (refer to Additional file 2:Test set for 2D-SAR). Additionally, after those inhibitors without IC50 values were filtered, which are not suitable for QSAR model. As a result, 63 inhibitors with pIC50 were kept as data set to building Topomer COMFA prediction model. The molecular structures of VEGFR-2 inhibitors along with their corresponding IC50 values were compiled to SDF file for the purpose of conducting a 3D-QSAR study (refer to Additional file 3:The molecular structures of VEGFR-2 inhibitors along with their corresponding pIC50).

In order to develop a prediction model for VEGFR-2 inhibitors, it was imperative to characterize the acquired compounds. Molecular descriptors serve as crucial tools in the fields of chemistry, pharmacology, environmental protection, and health research. Here, Discovery Studio 2020 software was utilized to generate a set of 160 molecular descriptors.

### Feature subset selection

The MRMR feature selection method, which is a filter selection method based on the mutual information maximization, was used to screening the molecule descriptors. The Maximum Relevance Minimum Redundancy (MRMR) feature selection method is based on the information theory concept of mutual information. Mutual information is a measure of the statistical dependence between two variables, indicating how much information one variable provides about the other [24, 25]. The MRMR method aims to select a subset of features that maximizes the relevance with the target variable while minimizing redundancy among the selected features. The underlying theory is that relevant features should have a strong relationship with the target variable, while redundant features should provide redundant or overlapping information that does not contribute significantly to the overall predictive power. The MRMR feature selection were performed by Expminer 2.0.

### Machine learning algorithms

Machine learning algorithms are increasingly being used to deal with the growth of huge data in the life sciences including drug design, protein prediction, epidemic prediction [26–44]. In this paper, ten machine learning algorithms including K nearest neighbor (KNN), Adaboost, Bagging, Random Forest (RF), Random Trees (RT), AD tree, C4.5, Bayes net, Support vector ma-chine(SVM) and Artificial neural network(ANN) were employed to building a prediction model for selecting the optimal VEGFR-2 inhibitors. All the parameters of each algorithms applied in this study could be referred to Additional file 5. The values of the parameters of the machine learning approaches. The brief theories of these algotithms are as following: The values of the parameters of the machine learning approaches.

### K nearest neighbor (KNN)

The K-Nearest Neighbors (KNN) algorithm is a supervised machine learning algorithm that can be used for both classification and regression tasks. It is a non-parametric method, meaning it does not make any assumptions about the underlying data distribution. At its core, the KNN algorithm operates on the principle of similarity. It assumes that data points with similar feature values are likely to have similar labels or outcomes. In other words, if a new data point is similar to its neighboring points, it is likely to belong to the same class or have a similar target value [45].

### Adaboost

Adaboost is a machine learning algorithm that belongs to the family of ensemble methods. It is primarily used for binary classification tasks, although it can be extended to handle multi-class problems as well. The main idea behind Adaboost is to iteratively train a series of weak classifiers on weighted versions of the training data. A weak classifier is a simple model that performs slightly better than random guessing. In each iteration, Adaboost adjusts the weights of misclassified samples, placing more emphasis on difficult-to-classify instances [46]. In this study.

### Bagging

Bagging is an integrated learning algorithm. It works by generating multiple weak learners and assembling them into an integrated prediction algorithm. When a prediction result is given, the integrated algorithm averages the results of the integrated multiple weak learners. When a category prediction is given, a plural vote is performed. The multiple prediction algorithms it contains originate from bootstrap replications of the learning sets and use these replication sets as new learning sets. Bagging’s weak learners are not correlated, but originate from random sampling. Since the sampling is random and samples are put back after sampling, there is a possibility of repeated sample collection [47].

### Random forest (RF)

Random Forest is an ensemble learning algorithm widely used in machine learning for classification and regression tasks. In Random Forest, a collection of decision trees is built using bootstrapping. Additionally, at each split in a tree, only a random subset of features is considered. For classification tasks, the final prediction is obtained through a voting mechanism, where each tree “votes” for the class label of a new data point, and the majority class label is assigned as the predicted label. In regression tasks, the final prediction is the average of the predicted values from all trees [48].

### Random trees (RT)

Random Tree, also known as Random Decision Tree, is a machine learning algorithm that is a variant of the popular decision tree algorithm. It combines the concepts of decision trees with randomization to create a more diverse and robust model. In a Random Tree, the construction process is similar to a traditional decision tree. It recursively splits the data based on different features and their thresholds to create a tree structure. However, there are two key differences that introduce randomness [49].

### C4.5

C4.5 is a decision tree algorithm developed by Ross Quinlan and widely used in machine learning for classification. It is an extension of the previous ID3 (Iterative Dichotomizer 3) algorithm, introducing several improvements and enhancements. C4.5 algorithm constructs a decision tree by recursively partitioning the training data according to the features that provide the greatest information gain. The aim is to create a tree that accurately predicts the class labels of instances based on their feature values [50].

### Bayes net

A Bayesian Network is a probabilistic graphical model that models dependencies and uncertainties among variables using directed acyclic graphs (DAGs). It assumes conditional independence between variables given their parent nodes. Each node has a conditional probability table (CPT) associated with it, representing the probability distribution given its parent node's state. Bayesian networks can be used for inference and prediction, allowing for inferences and predictions on unobserved variables. The structure and parameters of Bayesian networks can be learned from data containing known dependencies between variables and their states [51].

### Support vector machine (SVM)

Support Vector Machine (SVM) is a powerful supervised learning algorithm for classification and regression tasks. It is widely used to solve complex pattern recognition and decision boundary estimation problems. The basic principle of SVM is to find an optimal hyperplane for separating different classes of data points and maximizing the distance between the nearest classes of data points, which are called support vectors. The intuitive understanding of this approach is that by maximizing the bounds, SVM aims to achieve better generalization ability and robustness to unseen data [52].

### Artificial neural network (ANN)

Artificial Neural Network (ANN) is a computational model inspired by the structure and function of biological neural networks in the human brain. It is a powerful machine learning algorithm used for various tasks such as pattern recognition, classification, regression and optimization. The basic building blocks of artificial neural networks are artificial neurons, also known as nodes or perceptrons. Neurons receive inputs, apply weights to them, and produce outputs based on an activation function. These neurons are organized into a hierarchical structure that usually includes an input layer, one or more hidden layers, and an output layer. Information flows in the network in the forward direction, with each layer transforming the inputs until the final output is generated. During the training process, the connection weights between neurons are iteratively adjusted using optimization algorithms such as gradient descent. The goal is to minimize the difference between the predicted output and the actual output, thus improving the performance of the network [53].

### Topomer CoMFA

Topomer CoMFA is a virtual screening technique based on QSAR as proposed by Cramer [54, 55] which can be used as a Topomer technique and a CoMFA technique to overcome the associated alignment problems [56–58]. Topomer CoMFA has the advantage of simplicity, it can be modeled quickly and its modelling results are comparable to those of conventional CoMFA. In contrast to conventional CoMFA, Topomer CoMFA does not require manual stacking of ligands and can automatically stack regular Topomer. Instead, as with conventional CoMFA, stacking takes probe atoms to calculate electrostatic and steric fields and to model PLS. In the Topomer CoMFA method, the molecule is divided into different primitive fragments called “Topomers”. Each topomer represents a specific part of the molecule, similar to a functional group or moiety in a drug molecule. Subsequently, a three-dimensional molecular field is generated for each topomer, which describes various chemical properties within the molecule, such as steric configuration and charge distribution. These properties are used to construct a model describing the molecule-target interaction. Finally, using the collected activity data and molecular field information, a statistical analysis was performed to build a 3D-QSAR model related to biological activity. The model helps to predict the biological activity of other compounds. It is worth noting that Topomer CoMFA is a derivative of the CoMFA approach, and the core idea is to enhance the accuracy of prediction by modeling specific parts of the molecule. The exact methodology may vary depending on the study and the software tool [54, 59]. In this study, Topomer CoMFA were performed by Sybyl X2.0.

### Molecular docking

Molecular docking is a fundamental tool in the study of interactions between bio-logical molecules, based on the ‘lock and key model’ and the ‘induced-fit theory’ [60, 61]. The ‘lock and key’ model suggests that the matching of spatial shapes is the main requirement for distinguishing between different compounds. The two main topics of molecular docking methods are spatial matching and energy matching between molecules. Spatial matching is the basis for intermolecular interactions to occur, while energy matching is the basis for maintaining stable binding between molecules. Methods used for calculations regarding geometric matching include lattice point calculations, fragment growth, etc., while methods for energy calculations include simulated annealing, genetic algorithms, and so on. All of the above methods play a role in simplifying the system, and according to the degree of simplification as well as the way, they can be divided into the following three categories: rigid docking, semi-flexible docking and flexible docking. In this study, semi-flexible docking was applied. This method allows a certain degree of conformational change of the small molecules under study during the docking process, although the conformations of large molecules are generally fixed, and also restricts the adjustment of the conformations of small molecules, such as fixing the bond lengths and angles of certain non-critical parts [60]. Semi-flexible docking methods are more widely used among the various docking methods due to the amount of computation included as well as the predictive power of the model [62–68]. The steps of Semi-flexible docking are as following: firstly, a 2D small molecule database is constructed, secondly, the small molecules are processed according to the atom types and chemical bonding properties and converted into 3D structures and then saved; at the same time, the crystal structures of biological macromolecules are searched and downloaded through the Protein Crystal Structure Database (PDB) of the RCSB Protein Data Bank (RCSB) and the operations such as hydrogenation, charging, and energy minimisation are performed, combined with the establishment of pocket positions. At the same time, the crystal structure of the biomolecule was searched and downloaded from the PDB (RCSB Protein Data Bank), and hydrogenation, charge addition, and energy minimisation were performed. After the pocket position was established, the prepared small molecules were docked at the active pocket of the macromolecular receptor, and the conformations were optimized and interactions evaluated by a function; and the candidate molecules were filtered by a scoring function for future studies [69]. In this study, molecule docking was performed by Discover Studio 2020.

### Molecular dynamics simulation

Molecular Dynamics (MD) simulation is a computational technique employed to simulate the motion of atoms and molecules over a specified period. The fundamental principles underlying MD simulation are rooted in classical mechanics, quantum mechanics for atomic interactions, and the concepts of statistical mechanics. Molecular Dynamics simulation, by numerically solving Newton's equations of motion, allows for a detailed exploration of the structural, dynamic, and thermodynamic aspects of molecular systems. This method finds broad applications in biophysics, materials science, and drug design.

1. Potential Energy

The potential energy function is pivotal in molecular dynamics simulations, outlining intra- and inter-molecular interactions. It encompasses terms for bond, angle, dihedral angle energies, van der Waals forces, electrostatic forces, and charge-charge interactions. Parameters from this function seamlessly integrate into a force field, composed of mathematical expressions describing forces between atoms. This synergy forms the foundation for accurately modeling dynamic molecular behavior.

(2) Newton’s Equations of Motion

Newton's second law (F = ma)guides molecular dynamics, where F is the force from the force field, calculating acceleration (a) for each atom. This principle underlies molecular dynamics simulations, with numerical integration methods like Verlet or Leapfrog determining atomic velocity and position evolution over time.

(3) Initial and Boundary Conditions

Simulation begins with assigned initial positions and velocities, often stabilized by energy minimization. Boundary conditions, periodic or non-periodic, define interactions at system edges.

(4) Temperature and Pressure Control

Thermal bath algorithms, e.g., Nosé-Hoover thermostat, control temperature, while barostat algorithms manage pressure, crucial for realistic molecular environments.

(5) Simulation Time and Time Step

Simulation time, measured in femtoseconds or picoseconds, defines the overall duration, setting temporal scope. The time step, also in femtoseconds or picoseconds, governs numerical integration granularity, crucial for tracking atomic movements and determining computational efficiency.

(6) Dynamical Analysis

Examining energy profiles, velocity distributions, temperature, pressure, and other dynamic parameters refines understanding of molecular system dynamics.

## Results and discussion

### Feature selection and construction of 2D-SAR prediction model

To correctly determine an optimal VEGFR-2 inhibitor before structural modification and synthesis by using computer modeling programs would shorten the task of finding potentially useful drugs. Hence, in this study, a 2D-SAR prediction model was built to identify VEGFR-2 inhibitors. As the features affect the model’s prediction accuracy, maximum relevance-minimum redundancy (MRMR), which is a feature selection method, was applied, before the model was built. Generally, a greater number of descriptors can lead to improved statistical results and correlations. In QSAR studies, a guideline often suggests selecting the maximum number of descriptors while adhering to the principle that the descriptor count should not exceed 1/5 of the number of molecules in the training dataset [70]. It’s important to note that while increasing the number of descriptors can enhance the statistical outcomes, it's essential to balance this with the risk of overfitting and ensuring that the chosen descriptors are biologically relevant and meaningful in the context of the study's objectives. The decision to use a higher number of descriptors should be made in accordance with the specific goals of the research, the characteristics of the dataset, and the available domain knowledge.

**Fig. 1:**
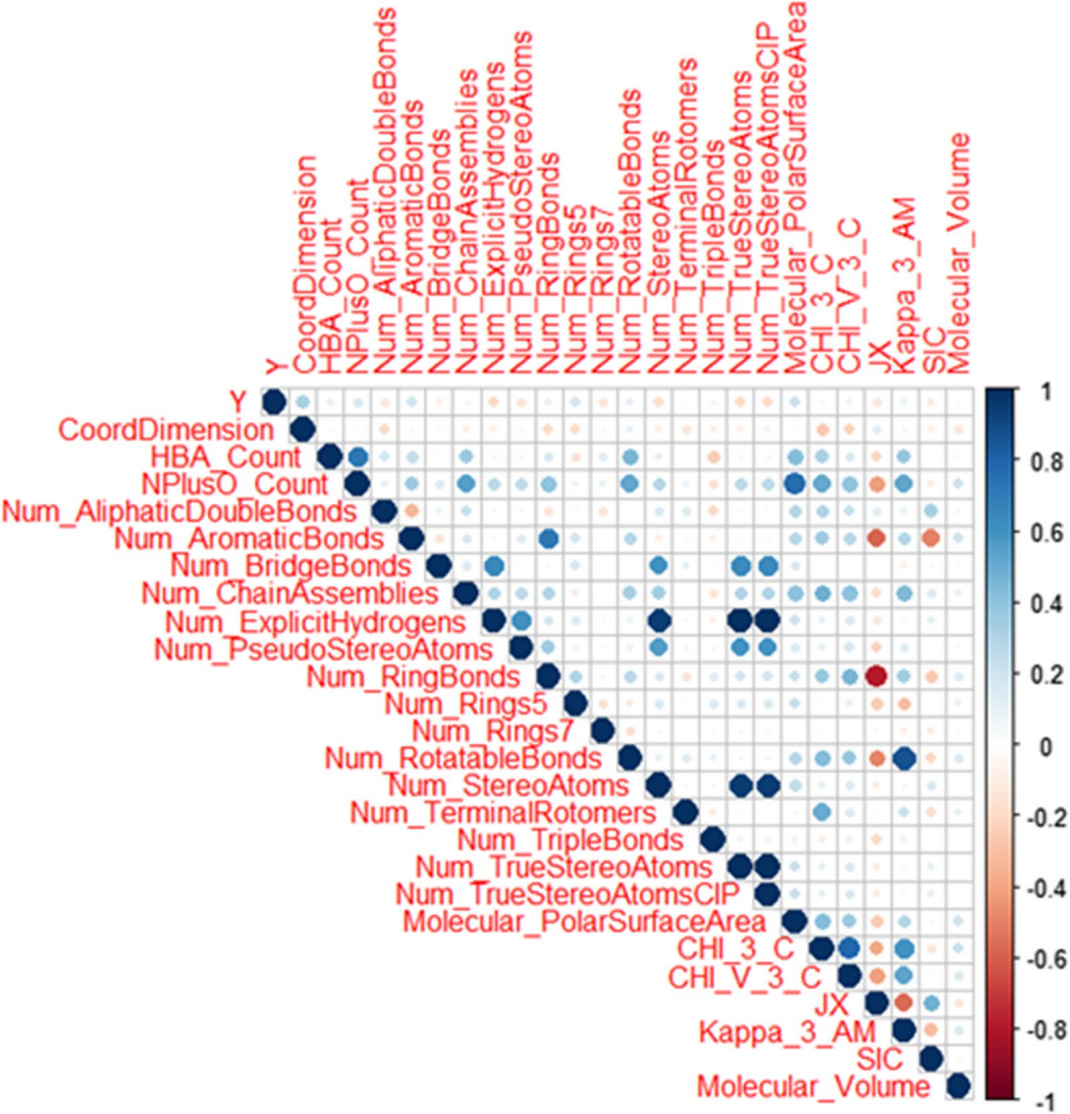
Correlation matrix of the 25 descriptors used

After screening the molecular descriptors with MRMR, a total of 25 molecule descriptors (the definitions see Additional file 4: Definitions of 25 descriptors) were obtained from the original 160 molecule descriptors (Fig. 1). Figure 1 shows that the correlation between the 25 descriptors and target is weak, which means that there is no strongly correlated descriptor, and of these CoordDimension and Molecular_PolarSurfaceArea are most correlated features.

Based on these 25 molecule descriptors, 10 machine learning approaches were used to build the prediction model (Table 1). The prediction ability of model was evaluated by tenfold cross-validation tests. From Table 1, it can be seen that the prediction accuracies with Adaboost, Random Forest and KNN are higher than 80% and the latter two achieved 87.3%. An independent set test was applied to further evaluate the model. The results also showed that the prediction accuracy of KNN achieved 84.3%, which was higher than the other nine machine learning algorithms. Hence, KNN was used to build the final prediction model due to its excellent prediction ability. Adaboost is a traditional ensemble learning algorithms, which has been applied widely in medicine science due to its capacity for over fitting [71, 72]. However, Adaboost is a time consuming algorithm due to the special form of sample selection and its classifier weighting [46]. Although KNN is a simple algorithm which judges unknown samples according to their clustering ability, as the prediction accuracy of KNN was the highest, it was selected to build the final prediction model. The prediction accuracy also showed that the combination of the molecule descriptors contributed to model building although singe descriptors were not correlated to the prediction.

**Table 1:** The prediction accuracy obtained by using different machine learning algorithms

Data set	Algorithm	SP	SN	ACC
Training Set	Adaboost	87.7	84.0	85.7
	Bagging	75.7	81.1	78.6
	RandomForest	88.5	86.2	87.3
	RandomTree	77.0	82.5	81.5
	C4.5	77.0	82.5	79.9
	ADTree	75.7	69.1	72.2
	KNN	85.2	88	87.3
	Bayes Net	80.7	69.5	74.7
	SVM	63.0	75.6	69.7
	ANN	79.8	86.5	83.4
Test Set	Adaboost	86.1	81.7	83.7
	Bagging	75.5	76.1	75.8
	RandomForest	85.4	79.4	82.2
	RandomTree	78.1	73.9	75.8
	C4.5	67.5	78.9	73.7
	ADTree	70.9	70.6	70.7
	KNN	82.1	83.9	84.3
	Bayes Net	78.1	58.7	67.1
	SVM	60.3	76.7	69.2
	ANN	76.8	80.6	78.9

For machine learning algorithm, optimizing parameters is an important issue. For example, for Support Vector Machine (SVM), parameters like Capacity (C), kernel choice, and gamma play key roles in accurately discerning inhibitor activity [73]. The Capacity parameter balances model complexity and generalization, sigma shapes the kernel function's behavior to capture both global trends and local variations, and gamma influences the decision boundary’s reach [74]. Through a systematic approach including grid search, cross-validation, and multiple metrics, optimal parameters were selected to enhance the SVM model's performance [75, 76]. Interesting, in this study, the results indicate that the simplest k-Nearest Neighbors approach yielded the best predictive outcomes. This phenomenon may be elucidated that nearest neighbor methods directly consider the samples in the training data that are most similar to the target samples, and thus may capture these features more accurately when the data has significant local similarity and density distribution. Moreover, complex models may suffer from dimensionality catastrophe when the features are of high dimensionality, whereas nearest neighbor methods are able to maintain a better generalization ability in high-dimensional spaces due to their local similarity-based approach. In addition, complex models are prone to overfitting problems on small sample data, whereas nearest neighbor methods may have an advantage in this regard due to fewer parameters. The nearest neighbor method is also relatively less susceptible to noise because it focuses on neighboring training samples. Furthermore, the complexity of the model may affect its performance, while the nearest neighbor method is suitable for small sample data as a relatively simple model. Finally, the simplest nearest-neighbor method achieves the best prediction results probably because it is more adapted to the data characteristics, has better generalization ability, and can effectively capture local similarities and distributions in the data while avoiding the problems faced by complex models, such as over-fitting and dimensionality catastrophe.

Machine learning (ML) models can serve as powerful screening tools to search databases such as Zinc, Binding DB, and NCI databases for potential drug candidates and predict the activity of database compounds. In this study, our model with KNN could be trained on a dataset of known active and inactive compounds against a specific biological target or activity, learning to correlate chemical features with biological responses. Once trained, KNN model can be applied to screen large compound libraries, such as those in Zinc, Binding DB, and NCI databases, to identify molecules with the highest likelihood of activity against the target of interest. Additionally, our prediction model can predict the activity or potency of database compounds based solely on their chemical structures, enabling rapid virtual screening and prioritization of compounds for experimental testing. For instance, our prediction model can be integrated into online prediction servers where users can input the chemical structure of a compound and receive a prediction of its activity against a specific target, providing a user-friendly platform for drug discovery and design.

### Construction of a Topomer CoMFA model

**Fig. 2:**
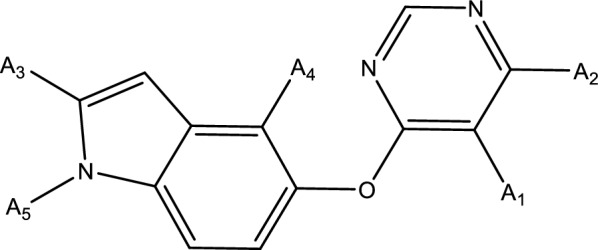
A structural representation of the compound template with the highest activity obtained. (A_1_:–NH_2_, A_2_:–HCNO–C_3_H_6_OH, A_3_:–CH_3_, A_4_:F,A_5_:–H)

Sixty-three inhibitor compound molecules together with their IC50 were collected from inhibitors to build a Topomer CoMFA model. The compound with the highest activity (Figure 2) was selected as the template. Two different cutting methods were chosen to build Topomer CoMFA model. Six principle components were applied y in modeling. The q^2^ values for models 1 and 2 were 0.678 and 0.309, respectively (see Table 2). The R^2^ values for models 1 and 2 were 0.899 and 0.508, respectively (see Table 2). As the q^2^ values of the model 1 was both greater than 0.5, this meant the established models were statistically significant (p < 0.05). Hence, model 1 was chosen to further study VEGFR-2 drug design and activity predictions. A cross-validation test was also performed to measure the prediction ability and experimental activity distribution of model 1 (Table 3).

**Table 2:** The results from the two Topomer CoMFA model studies

Dataset	Model 1	Model 2
Cutting model		
q^2^	0.678	0.309
R^2^	0.899	0.508

**Table 3:** The predicted and actual pIC50 of compounds for model 1

NO.	pIC50		NO.	pIC50		NO.	pIC50	
	Actual	Predict		Actual	Predict		Actual	Predict
*1*	6.30	6.31	*22*	6.98	7.05	*43*	6.41	5.88
*2*	6.00	6.14	*23*	6.94	6.24	*44*	6.4	6.60
*3*	6.41	6.39	*24*	6.93	6.41	*45*	6.38	7.03
*4*	6.72	6.72	*25*	6.92	7.25	*46*	6.3	6.11
*5*	6.77	6.98	*26*	6.91	6.85	*47*	6.3	7.49
*6*	6.87	6.74	*27*	6.89	6.13	*48*	6.29	7.48
*7*	6.6	6.82	*28*	6.87	5.85	*49*	6.25	6.64
*8*	6.58	6.81	*29*	6.84	6.87	*50*	6.23	6.24
*9*	6.52	6.37	*30*	6.77	6.03	*51*	6.21	6.63
*10*	7.37	7.40	*31*	6.74	7.22	*52*	6.15	6.27
*11*	7.12	6.89	*32*	6.73	6.81	*53*	6.15	7.37
*12*	7.08	6.78	*33*	6.72	7.63	*54*	6.13	6.52
*13*	6.94	7.15	*34*	6.7	6.22	*55*	6.12	6.56
*14*	6.25	6.45	*35*	6.67	7.51	*56*	6.00	6.16
*15*	6.30	6.25	*36*	6.63	7.48	*57*	6.00	6.08
*16*	6.93	6.70	*37*	6.60	7.02	*58*	5.98	6.981
*17*	7.28	7.31	*38*	6.58	7.28	*59*	5.98	6.77
*18*	7.62	7.37	*39*	6.56	6.68	*60*	5.94	6.11
*19*	7.03	7.12	*40*	6.56	6.37	*61*	5.92	5.99
*20*	7.38	7.41	*41*	6.52	7.37	*62*	5.87	6.88
*21*	5.92	5.81	*42*	6.46	5.93	*63*	5.8	6.58

**Fig. 3:**
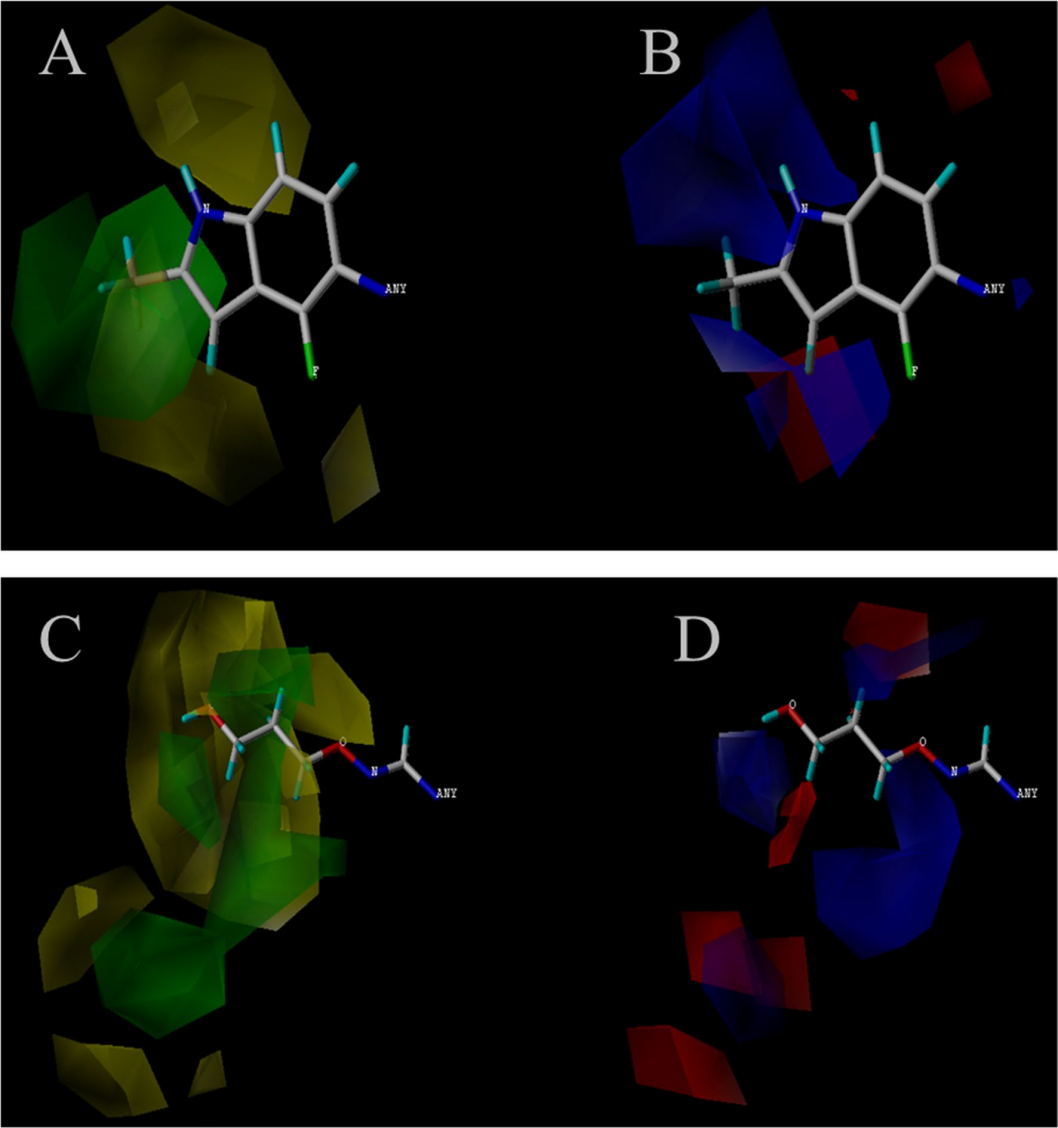
3D contour maps of the Topomer CoMFA model for compound 47. **A** represent the steric contour maps of R1, **B** represent the electrostatic field maps of R1, **C** represent the steric contour maps of R2, **D** represent the electrostatic field maps of R2

Besides well quantitatively prediction, Topomer CoMFA model also provide relevant field information. Model 1 showed steric and electrostatic contour maps for the R1 and R2 sub-structure groups. The compound with highest activity was selected as an example to analyze (see Fig. 3). Green, yellow, blue and red represent adding large volume groups, small volume groups, positive charged groups and negative-charged groups, respectively, which can enhance the compound’s activity. Hence, increasing electrostatic field by adding big group at A_3_ will benefit high activity and vice versa. Meanwhile, small groups with a positive-charge may also increase the compound’s bioactivity in A2 group. Negatively charged groups such as –F,–Br, will improve the activity at A4. Positively charged groups such as CH3^+^ would increase the activity at A5. Large groups such as sulfonic acid group can also be introduced at A4, which may improve the drug molecular activity.

Here, although we collected over 600 chemical compounds, only a subset of compounds had PIC50 values. Therefore, after screening, we obtained a final set of 63 VEGFR-2 inhibitor molecules with pIC50 values. Through the established model, we observed that the model's predictive accuracy meets our requirements and is suitable for research purposes. However, we are aware that larger datasets contribute to more stable models. Therefore, we will continue to focus on relevant chemical compounds and update our dataset in the future.

### Interactions between inhibitors and VEGFR-2

**Fig. 4:**
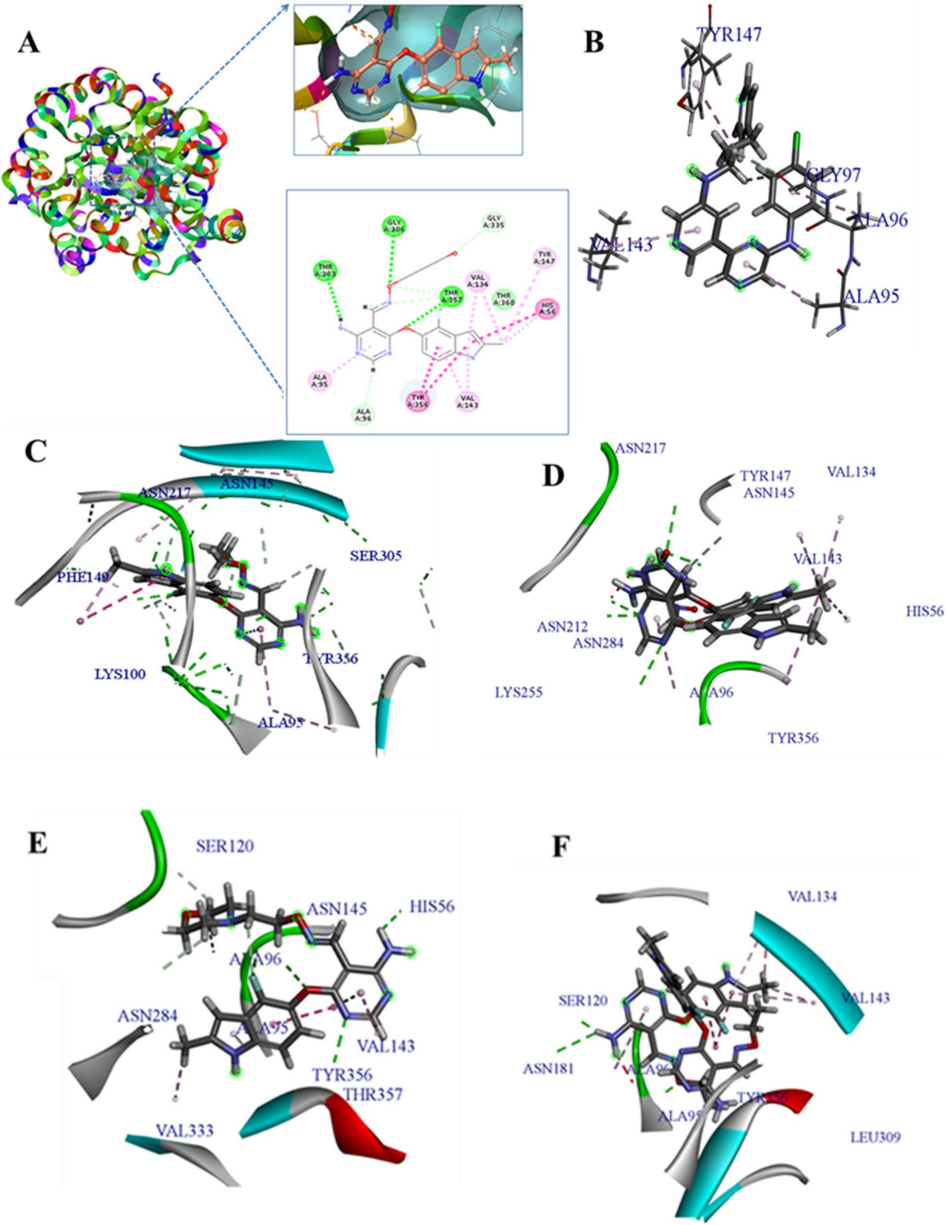
Interaction diagrams of the compounds and their acceptor (6ET4). **A**. The location of the active pocket with the upper right three-dimensional diagram showing the docking target, and the lower right two-dimensional diagram showing the docking effect. **B**–**F** The binding sites of compounds 1–5 with VEFGR-2

Here, top five activity molecules (compounds 1–5) were selected to investigate their interactions to VEGFR-2 by using molecular docking. The results showed that these five molecules could all interact with VEGFR-2 with high docking scores (Table 4). The docking scores of the five top activity molecules were all higher than the five low activity molecules. We also found that some molecules bound to VEGFR-2 via hydrogen bonds at ASN217, ASN145, SER305, ASN284 and LYS255. In addition, some molecules also shared VAL143,VAL134,TYR356 and ALA96 in order to bind to VEGFR-2 with hydrophobic forces (Fig. 4). The interactions of the five poor activity molecules and VEGFR-2 were also studied. After simulation, the results showed that the high activity inhibitors could always bind to VEGFR-2 in a more stable way than the poor activity inhibitors, although there was no well correlation between the pIC50s and their docking scores. The docking scores of compounds 59–63 were all significantly lower than compounds 1–5 which suggested the interactions with the former molecules were unstable with poor activities.

**Table 4:** The results of molecular docking of ten compounds

NO	pIC50	Total score(Kcal/mol)
1	7.96	131.28
2	7.62	116.68
3	7.49	118.74
4	7.48	105.96
5	7.41	102.60
59	5.68	64.31
60	5.64	55.72
61	5.92	53.55
62	5.87	75.27
63	5.8	78.34

**Fig. 5:**
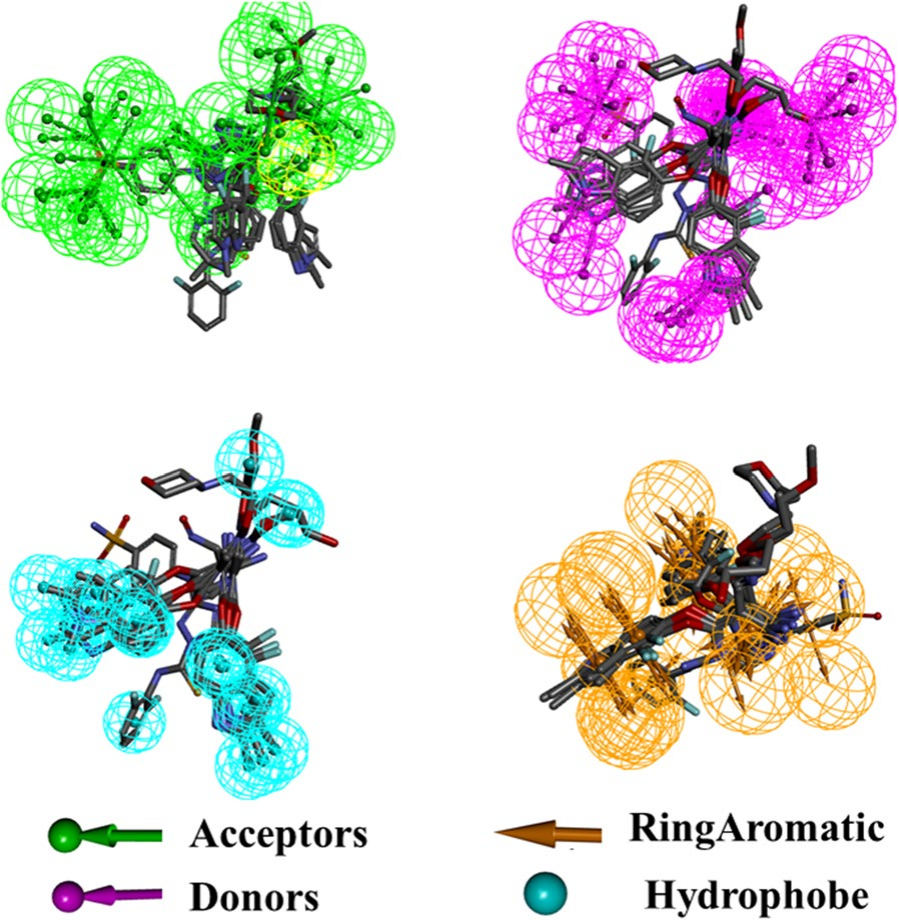
The pharmacophore features of the inhibitor compounds

In order to explore the relationship of activity and hydrogen bonds/ hydrophobic forces, the pharmacophore of VEGFR-2 inhibitors were studied. As a result, four features of pharmacophores were obtained including H-acceptors, H-donors, Hydrophob and Ring aromatic which may affect greatly the activities of compounds (Fig. 5). Figure 5 suggested that the two aromatic rings contribute to the hydrophobic forces of the compounds. However, the NO- and NH- groups can also provide donors and acceptors to form hydrogen bonds which can improve the activities of the inhibitors.

The ‘Lock and key model’ and the ‘induced-fit theory’ are the basis for the docking of molecules [60]. When ligands and receptors bind to each other, there are electrostatic, hydrogen bonding and van der Waals force interactions as well as hydrophobic forces involved. The binding of a ligand and a receptor must satisfy the principle of mutual matching, i.e., their geometry and electrostatic, hydrogen bonding and hydrophobic interactions must complement each other. In this study, the molecule-receptor complexes with high pic50 always had strong interaction forces such as hydrogen bonds and electrostatic interactions that satisfied Lipinski’s theory [77]. However, bulky and negative groups are the key factors for high activities of VEGFR-2 inhibitors which is similar to Tong’s study [78].

In the reversible interaction of ligand with VEGFR-2, ligand-receptor binding is transient and can be disrupted or reversed. At this point, the ligand interacts with specific binding sites on the receptor through non-covalent interactions such as hydrogen bonding, van der Waals forces and hydrophobic interactions [79, 80]. In contrast, the irreversible interaction between ligand and VEGFR-2 involves the formation of a covalent bond between the ligand and the receptor. The formation of this covalent bond results in a permanent or long-lasting connection between the ligand and the receptor that cannot be easily reversed. Irreversible binding typically occurs when the ligand contains a reactive functional group that can form covalent bonds with specific amino acid residues in the receptor binding site [81]. Such covalent modifications can lead to irreversible inhibition or activation of receptor function. It is important to note that the distinction between reversible and irreversible interactions is not always absolute. Some interactions may exhibit characteristics of both reversible and irreversible binding, depending on factors such as the concentration and duration of exposure to the ligand [82].

### Design of potential VEGFR-2 inhibitors

In order to optimize the activity of the VEGFR-2 inhibitors, we conducted molecular design and structural modifications based on the information provided by the 2D/3D-QSAR models and pharmacophore features regarding the R1 and R2 substructural groups. The rational explanation for the design of the compounds in this text is based on a multi-faceted approach to optimizing the activity of VEGFR-2 inhibitors. The approach includes several structural modifications informed by 2D/3D-QSAR models and pharmacophore features, particularly focusing on the R1 and R2 substructural groups. Here are the main idea of our rational design:

1. Introduction of Small Amino Groups at Position A2. The incorporation of small amino groups with positive charges at position A2 is aimed at enhancing bioactivity. The positive charge can potentially improve the interaction of the compound with the target receptor. Adjusting the charge density at this position is a way to fine-tune the interaction further.
2. Introduction of Negatively Charged Groups at Position A4. At position A4, negatively charged groups like -F and -Cl were introduced to enhance the compound's activity. These groups can potentially form strong interactions with specific regions of the receptor, leading to improved inhibitory effects.
3. Incorporation of Aromatic Heterocycles. Aromatic heterocycles were considered to enhance hydrophilicity, which can be advantageous in terms of increasing the compound's activity. Different combinations of charges and sizes of these heterocycles were explored to optimize the interactions with the target receptor. Retention of Two Aromatic Rings: Retaining two aromatic rings in the compound's structure is intended to augment hydrophobic interactions. This feature is important for enhancing the binding of the inhibitor to the receptor, which can lead to improved activity.
4. Introduction of NH-Groups for Hydrogen Bonding. NH-groups were introduced to provide both donors and acceptors for hydrogen bonding. This feature improves the compound's ability to form specific interactions with the receptor, potentially increasing its inhibitory activity.

The overall design strategy is to leverage information from computational models (2D/3D-QSAR models), understand the pharmacophore features relevant to the receptor (H-acceptors, H-donors, hydrophobicity, and ring aromaticity), and make targeted structural modifications to enhance the compound’s activity. As a result of this rational design approach, five potential VEGFR-2 inhibitors were obtained, which were predicted to have the potential to inhibit VEGFR-2 to a greater extent based on their structure and predicted pIC50 values. The structure of molecules and predicted pIC50 were listed in Table 5.

**Table 5:** Predicted pIC50 of designed VEGFR-2 inhibitors

NO	Structure	Predicted pIC50
1		8.30
2		8.29
3		8.17
4		8.02
5		8.00
6		8.00
7		8.00
8		7.94
9		7.87

**Fig. 6:**
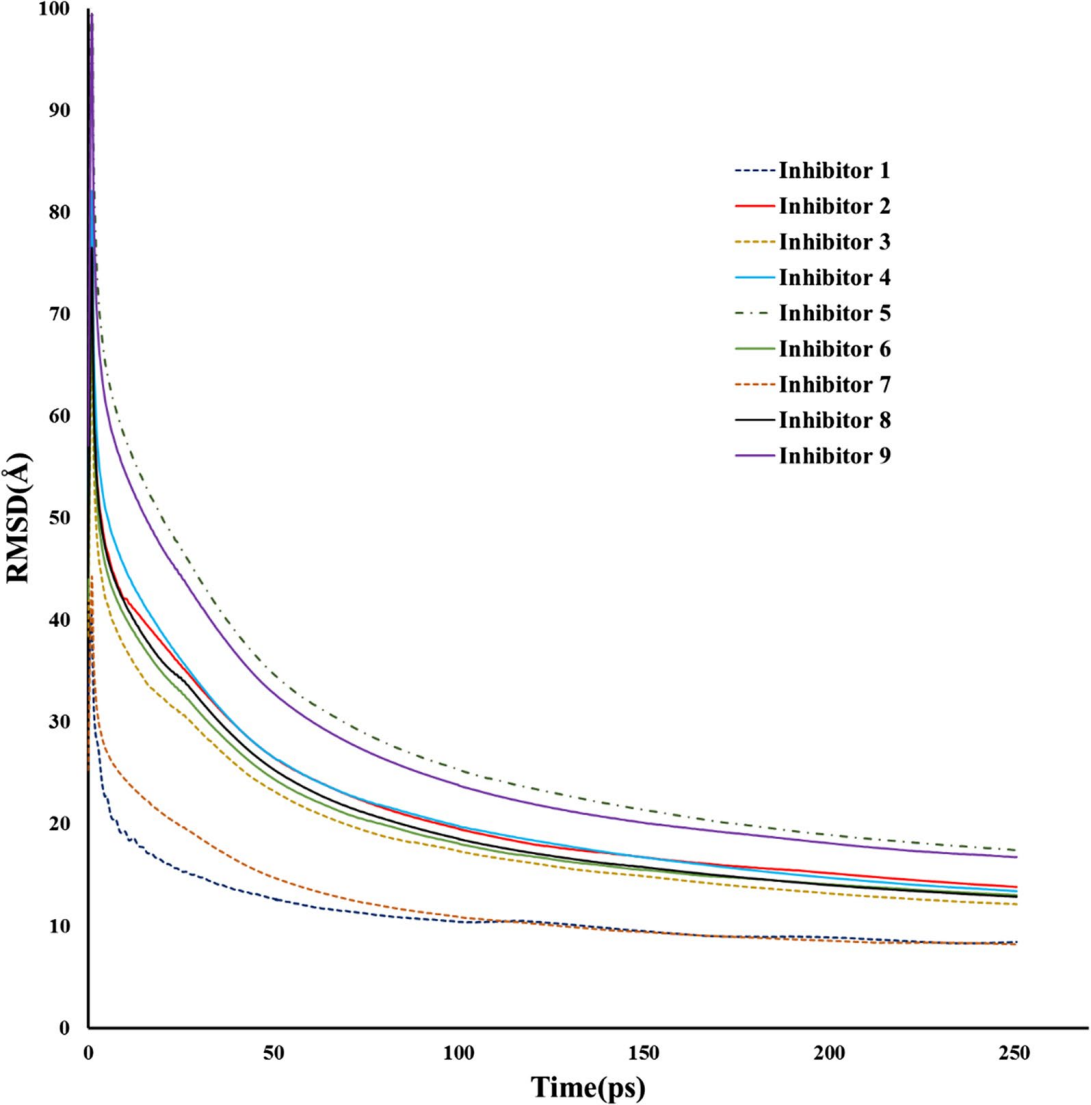
RMSD observed during MD simulation of 250 ps for the VEGFR-2 complex of designed inhibitors

We also performed molecular dynamics (MD) studies for the final designed inhibitors to gain a comprehensive understanding of their behavior in complex biological environments. We conducted 250 ps molecular dynamics (MD) simulations for each designed inhibitor- VEGFR-2 complex systems in an explicit aqueous solution. To assess the stability of each designed inhibitor within VEGFR-2 and validate the credibility of the MD simulation outcomes, we examined the root mean square deviation (RMSD) across the 250 ps MD trajectories. RMSD serves as a metric to characterize the temporal disparity between the VEGFR-2 and the initial structure, acting as an indicator of whether the system has attained a state of kinetic equilibrium. Figure 6 depicts the RMSD curves for each complex throughout the 250 ps MD simulation. As illustrated, designed inhibitors progressively achieves a state of kinetic equilibrium after the initial fluctuations. For the nine designed inhibitors, the systems of gradually reached MD equilibrium in the last 20 ps. Notably, for inhibitor 1 and 7, the fluctuations typically ranged between 8.3 and 8.4 from 228 ps. The equilibrium state in the initial 50 ps indicated that the initial conformation of the designed inhibitors and its binding mode with VEGFR-2 were unstable. However, stability was eventually achieved in the last 50 ps. All systems achieved molecular dynamics equilibrium within the final 20 ns. It is important to highlight that inhibitors 5 and 9 exhibited higher fluctuations during the final 20 ns, suggesting potential flexibility or dynamic behavior in their binding profiles compared to other inhibitors.

Molecular dynamics simulations offer a dynamic perspective, capturing the intricate motions and conformational changes that molecules undergo over time. This approach provides valuable insights into the stability of binding modes between the designed analogues and their target proteins, shedding light on the robustness of these interactions. In this study, we performed MD for designed inhibitors by considering the influence of the solvent environment. The results indicate that the designed inhibitors undergo dynamic structural changes during the simulation, eventually settling into stable binding conformations with VEGFR-2. The analysis of RMSD provided insights into the temporal disparity between the initial and final structures, confirming the attainment of a state of kinetic equilibrium for all designed inhibitors. This information is crucial for understanding the stability and behavior of these inhibitors in a realistic biological context, aiding in the assessment of their potential as therapeutic agents targeting VEGFR-2. The study’s rigorous approach to MD simulations and detailed analysis enhances the credibility of the findings and contributes valuable data to the field of drug design and molecular interactions in complex biological systems.

VEGFR-2 inhibitors can modulate the signaling pathways associated with VEGFR-2, leading to various effects on angiogenesis and related processes. Several key signaling pathways can be influenced by VEGFR-2 inhibitors such as VEGF/VEGFR Signaling Pathway, PI3K/Akt/mTOR Pathway and MAPK/ERK Pathway. The binding of VEGF to VEGFR-2 activates downstream signaling cascades, including the PI3K/Akt and MAPK/ERK pathways. VEGFR-2 inhibitors can block these pathways by preventing the binding of VEGF to the receptor, thereby inhibiting angiogenesis [83]. Meanwhile, VEGFR-2 activation leads to the activation of PI3K, which in turn activates protein kinase B (Akt) and mammalian target of rapamycin (mTOR) [84–86]. These signaling molecules play crucial roles in cell survival, proliferation, and angiogenesis. Moreover, VEGFR-2 activation triggers the activation of MAPK/ERK signaling, which contributes to angiogenic processes. VEGFR-2 inhibitors can disrupt this pathway by inhibiting the activation of VEGFR-2 and downstream signaling [87, 88]. By targeting VEGFR-2 and modulating these signaling pathways, VEGFR-2 inhibitors have potential therapeutic applications in conditions characterized by excessive angiogenesis, such as cancer, age-related macular degeneration, and certain inflammatory disorders. These inhibitors can help suppress abnormal blood vessel formation and inhibit the growth and spread of tumors by interfering with the signaling cascades driven by VEGFR-2 activation.

In addition, the toxicity of VEGFR-2 inhibitors is an important consideration in drug development and therapeutic applications. VEGFR-2 is a receptor involved in angiogenesis, the process of forming new blood vessels. Inhibiting VEGFR-2 can have both therapeutic benefits and potential adverse effects. While VEGFR-2 inhibitors have shown promise in anti-cancer therapies and the treatment of other diseases, they can also be associated with certain toxicities including cardiovascular toxicity, wound healing impairment, gastrointestinal toxicity and hepatotoxicity. While the toxicity of VEGFR-2 inhibitors can be influenced by various factors, including the overall molecular structure and pharmacokinetic properties, there are certain substructures that have been associated with potential toxicity such as electrophilic functional groups, aromatic or heterocyclic rings with high lipophilicity, quinone-like structures and nitro-aromatic compounds. However, in this study, these substructures also may increase the activity of the inhibitors. As the overall toxicity of a compound is influenced by multiple factors and can be context-dependent, careful consideration of these substructures during the design and optimization of VEGFR-2 inhibitors can help guide the identification and modification of potentially toxic elements in the molecule. Additionally, it is crucial to perform comprehensive toxicity assessments and preclinical studies to evaluate the safety profile of VEGFR-2 inhibitors and identify potential adverse effects during the drug development process. Hence, more QSPR prediction models should be developed to further assist the molecule design for VEGFR-2 inhibitors.

In contrast to previous modeling techniques applied to VEGFR-2 inhibitors, our study distinguishes itself by employing a dataset of 600 compounds with diverse structures. Unlike previous studies that often focused on a smaller set of compounds with similar structures, our approach encompasses a broad range of chemical diversityFor instance, Fariba et al. utilized naïve ANN methods to discover VEGFR2 inhibitors for 33 compounds [89]. Similarly, Sobhy et al., Merve et al., El-Gazzar et al., Sun et al. employed 3D-QSAR pharmacophore and docking modeling to identify a novel scaffold for inhibiting VEGFR2 based on seriouses compounds [90–93].

Our study's emphasis on utilizing a diverse compound set allows for a comprehensive exploration of chemical space and a broader coverage of potential VEGFR-2 inhibitors. This approach enhances the generalizability and applicability of our findings, as it encompasses a wider range of structural motifs and chemical properties. Our study’s utilization of a diverse compound set represents a departure from previous methodologies, offering innovative insights into the structural activity relationships of VEGFR-2 inhibitors. By encompassing a broad spectrum of chemical structures, our approach expands the scope of VEGFR-2 inhibitor discovery and holds promise for the development of novel therapeutics with improved efficacy and safety profiles.

## Conclusions

QSAR research plays an important and widely used modern drug design methods. SAR studies can quickly screen target compounds, thus saving a lot of time and money. In this study, we established 2D-SAR and 3D-QSAR predictive models for designing potential VEGFR-2 inhibitors. Several modeling methods based on the molecular descriptor and three-dimensional structure of novel compounds were used. Five potentially useful compounds were obtained and these will aid in the search for novel VEGFR-2 inhibitors for the treatment of patients with liver cancer.

### Supplementary Information

**Additional file 1.** Training set for 2D-SAR.

**Additional file 2.** Test set for 2D-SAR.

**Additional file 3.** The molecular structures of VEGFR-2 inhibitors along with their corresponding pIC50.

**Additional file 4.** Definitions of 25 descriptors.

**Additional file 5.** The values of the parameters of the machine learning approaches.

## Data Availability

Datasets are available from the corresponding author on reasonable request.
