# Supplementary material for: QSAR analysis of VEGFR-2 inhibitors based on machine learning, Topomer CoMFA and molecule docking
Source: BMC Chem. 2024 Mar 30;18(1):59. doi: 10.1186/s13065-024-01165-8 (PMC10981835; doi:10.1186/s13065-024-01165-8)
Supplement: Supplementary file 4 — Additional file 4. Definitions of 25 descriptors. [file 13065_2024_1165_MOESM4_ESM.docx]

Definitions of 25 descriptors

| NO | Name | Definition |
| --- | --- | --- |
| 1 | CoordDimension | Coordinate dimensions of the molecule, typically referring to its spatial extent along the X, Y, and Z axes in three-dimensional space. |
| 2 | HBA_Count | Number of hydrogen bond acceptors in the molecule, representing atoms capable of accepting hydrogen bonds. |
| 3 | NPlusO_Count | Number of nitrogen and oxygen atoms in the molecule. |
| 4 | Num_AliphaticDoubleBonds | Number of aliphatic double bonds in the molecule. |
| 5 | Num_AromaticBonds | Number of aromatic bonds in the molecule. |
| 6 | Num_BridgeBonds | Number of bridge bonds in the molecule, which connect different parts of a molecular structure. |
| 7 | Num_ChainAssemblies | Number of chain assemblies in the molecule. |
| 8 | Num_ExplicitHydrogens | Number of explicitly defined hydrogen atoms in the molecule. |
| 9 | Num_PseudoStereoAtoms | Number of pseudo-stereocenters in the molecule. |
| 10 | Num_RingBonds | Number of bonds in ring structures within the molecule. |
| 11 | Num_Rings5 | Number of five-membered rings in the molecule. |
| 12 | Num_Rings7 | Number of seven-membered rings in the molecule. |
| 13 | Num_RotatableBonds | Number of rotatable bonds in the molecule, representing single bonds that can rotate freely. |
| 14 | Num_StereoAtoms | Number of stereoatoms in the molecule. |
| 15 | Num_TerminalRotomers | Number of terminal rotomers in the molecule. |
| 16 | Num_TripleBonds | Number of triple bonds in the molecule. |
| 17 | Num_TrueStereoAtoms | Number of true stereoatoms in the molecule. |
| 18 | Num_TrueStereoAtomsCIP | Number of true stereoatoms using Cahn–Ingold–Prelog (CIP) notation. |
| 19 | Molecular_PolarSurfaceArea | Polar surface area of the molecule, representing the surface area occupied by polar atoms. |
| 20 | CHI_3_C | Third-order connectivity index for carbon atoms. |
| 21 | CHI_V_3_C | Third-order valence connectivity index for carbon atoms. |
| 22 | JX | JX descriptor, representing a specific property or characteristic of the molecule. |
| 23 | Kappa_3_AM | Kappa shape index for the molecule. |
| 24 | SIC | Shape index of the molecule. |
| 25 | Molecular_Volume | Volume occupied by the molecule. |
