# Supplementary material for: QSAR analysis of VEGFR-2 inhibitors based on machine learning, Topomer CoMFA and molecule docking
Source: BMC Chem. 2024 Mar 30;18(1):59. doi: 10.1186/s13065-024-01165-8 (PMC10981835; doi:10.1186/s13065-024-01165-8)
Supplement: Supplementary file 5 — Additional file 5. The values of the parameters of the machine learning approaches. [file 13065_2024_1165_MOESM5_ESM.docx]

The values of the parameters of the machine learning approaches

| Algorithm | Basic classifier | Parameters |
| --- | --- | --- |
| Adaboost | Randomforest, | weightThreshold=100, numIterations=10 |
| Bagging | Fast decision tree | learner,bagSizePercent=100, |
| RandomForest | - | numTrees =10 |
| RandomTree | - | minNum=1.0 |
| C4.5 | - | confidenceFactor=0.25 |
| ADTree | - | numOfBoostingIterations=10 |
| KNN | - | K=1 |
| Bayes Net | - | Alpha=0.5 |
| SVM | - | Kernal=polykernel, C=1 |
| ANN | - | learningRate=0.3;  hiddenLayers=a; momentum=0.2;training time =5000 |
